# Treatment of amblyopia in the adult: insights from a new rodent model of visual perceptual learning

**DOI:** 10.3389/fncir.2014.00082

**Published:** 2014-07-16

**Authors:** Joyce Bonaccorsi, Nicoletta Berardi, Alessandro Sale

**Affiliations:** ^1^Department of Medicine, Institute of Neuroscience CNR, National Research Council (CNR)Pisa, Italy; ^2^Department of Psychology, Florence UniversityFlorence, Italy

**Keywords:** amblyopia, visual acuity, environmental enrichment, perceptual learning, GABAergic inhibition

## Abstract

Amblyopia is the most common form of impairment of visual function affecting one eye, with a prevalence of about 1–5% of the total world population. Amblyopia usually derives from conditions of early functional imbalance between the two eyes, owing to anisometropia, strabismus, or congenital cataract, and results in a pronounced reduction of visual acuity and severe deficits in contrast sensitivity and stereopsis. It is widely accepted that, due to a lack of sufficient plasticity in the adult brain, amblyopia becomes untreatable after the closure of the critical period in the primary visual cortex. However, recent results obtained both in animal models and in clinical trials have challenged this view, unmasking a previously unsuspected potential for promoting recovery even in adulthood. In this context, non invasive procedures based on visual perceptual learning, i.e., the improvement in visual performance on a variety of simple visual tasks following practice, emerge as particularly promising to rescue discrimination abilities in adult amblyopic subjects. This review will survey recent work regarding the impact of visual perceptual learning on amblyopia, with a special focus on a new experimental model of perceptual learning in the amblyopic rat.

## Amblyopia

### Definition and peculiarities of the disorder

Amblyopia (from the Greek, *amblyos*-blunt; *ops*-vision), also called “lazy eye”, is a developmental abnormality usually associated with physiological alterations in the visual cortex occurring early in life (Ciuffreda et al., 1991; Holmes and Clarke, 2006). In humans, this pathology occurs approximately in 1–5% of the population, and is generally associated with an early history of abnormal visual experience due to binocular misalignment (strabismus), image degradation (high refractive error and astigmatism and anisometropia), or form deprivation (congenital cataract and ptosis). The rare amblyogenic condition called congenital or early-acquired media opacity causes a form of amblyopia called deprivation amblyopia, the most severe and damaging type of amblyopia. In this case, cataracts, corneal lesions, or ptosis block or distort retinal image formation.

Regardless of its etiology, amblyopia is usually unilateral: visual acuity of one eye is reduced with respect to the other eye. Associated symptoms include poor stereoscopic depth perception, and low contrast sensitivity and reduced motion sensitivity in the weaker eye. In the clinical setting, however, the damage produced by amblyopia is generally expressed as a loss of visual acuity in an apparently healthy eye, despite appropriate optical corrections.

In contrast with early investigations indicating the retina as the primary site of amblyopia (Hess, 2001), many studies have confirmed that the retina exhibits normal physiology in amblyopic subjects (Sherman and Stone, 1973; Kratz et al., 1979; Baro et al., 1990); the lateral geniculate nucleus of the thalamus (LGN), instead, appears to be to some extent affected be sensory deprivation in one eye, with some cells exhibiting less than normal peripheral suppression and with a profound atrophy in the geniculate layers receiving inputs from the deprived eye (Wiesel and Hubel, 1963). The current consensus, however, is that amblyopia mostly originates from alterations in neural circuitries in the primary visual cortex (V1; Levi and Harwerth, 1978; Blakemore and Vital-Durand, 1986; Hess, 2001; Barrett et al., 2004), due to a combination of altered visual experience and high neuronal plasticity in the cortical developing circuits.

Development of visual system circuits depends on the interaction between genetic programs and experience-driven plasticity processes (Goodman and Shatz, 1993; Katz and Shatz, 1996), the latter being required for a proper refinement of neural circuits (Weliky, 2000; Lewis and Maurer, 2009). Critical periods (CPs) are time windows in early postnatal life during which plasticity is enhanced and neural circuits display a heightened sensitivity to acquire instructive and adaptive signals from the external environment. CPs for experience-dependent plasticity are widespread in the animal kingdom (Berardi et al., 2000), and have been demonstrated not only for the visual, auditory and somatosensory systems, but also for cognitive functions, including acquisition of song in birds and language in humans (Doherty, 1997; Doupe and Kuhl, 1999; Berardi et al., 2000; Hensch, 2004).

It is now clear that there are different CPs not only for different functions (even within the same sensory system; e.g., Harwerth et al., 1986, 1990), but also for different parts of the brain (even within different layers of V1; LeVay et al., 1980), and distinct CPs for recovery from and for induction of sensory deprivation effects (Berardi et al., 2000). The CP is not a simple, age-dependent maturational process, but is rather a series of critical developmental events controlled in a use-dependent manner. In agreement with this concept, a total absence of sensory inputs leads to a delay in the functional and anatomical maturation of the visual system. For example, the visual cortex of animals reared in darkness from birth (dark rearing, DR) displays prominent physiological deficits, including reduced orientation and direction tuning, lower cell responsiveness and increased latency, larger receptive field (RF) sizes, altered spontaneous activity, rapid habituation to repeated stimulus presentation, immature ocular dominance (OD) distribution and lower visual acuity (Frégnac and Imbert, 1978; Timney et al., 1978; Benevento et al., 1992; Fagiolini et al., 1994; Pizzorusso et al., 1997). Moreover, animals reared from birth in complete darkness have a delayed CP time course, with abnormal levels of plasticity persisting into adulthood (Mower, 1991; Fagiolini et al., 1994; Iwai et al., 2003).

The CP for the development of amblyopia closes around 6–8 years of age in humans (Worth, 1903; von Noorden, 1981). Alterations in visual experience caused by strabismus or high anisometropia with onset beyond this age do not result either in the severe loss of visual acuity for the affected eye or in the severe reduction in binocular vision caused by altered visual experience with an earlier onset. What is more important, however, is that if the correction of strabismus or anisometropia is delayed past this age, recovery of visual acuity and binocular vision is almost absent; indeed, the magnitude of the recovery is progressively reduced as the corrective intervention is made at progressively increasing ages during childhood, with negligible recovery obtained after 8 years of age. That is, in addition to the occurrence of a CP for the establishment of amblyopia, there is also a sensitive period for a successful treatment of this pathology (see Lewis and Maurer, 2009).

### Neural mechanisms underlying amblyopia

Much of our current understanding of the neural mechanisms underlying amblyopia derives from studies on animal models, revealing that major pathological changes in this pathology occur at the cortical level.

In animal models, amblyopia can be easily induced by imposing a reduction of inputs from one eye by lid suture (monocular deprivation, MD) during the CP. This treatment dramatically decreases V1 binocularity, shifting the physiological responsiveness of visual cortical neurons towards the open eye. As a direct consequence, the visual acuity of the deprived eye is strongly reduced and its contrast sensitivity is blunted (Wiesel and Hubel, 1963; Hubel and Wiesel, 1970; Olson and Freeman, 1975; Movshon and Dürsteler, 1977; Olson and Freeman, 1980). In their pioneering experiments, Hubel and Wiesel observed that, in kittens, the susceptibility to the effects of MD starts suddenly near the beginning of the fourth week of life, remains robust between the sixth and eighth weeks, and then declines completely after the third month, thus defining a CP for MD effectiveness. MD starting in adulthood produced no detectable outcome (Hubel and Wiesel, 1970; Olson and Freeman, 1980). The effects of MD and the existence of a CP for OD plasticity have been subsequently described also in several other species of mammals (Van Sluyters and Stewart, 1974; Hubel et al., 1977; Blakemore et al., 1978; LeVay et al., 1980; Emerson et al., 1982; Fagiolini et al., 1994; Horton and Hocking, 1997; Issa et al., 1999). While the effects of MD can be reversed to a limited extent during the CP by reversing the condition of visual deprivation, the same deficits become irreversible later on (Wiesel and Hubel, 1965; Movshon, 1976; Van Sluyters, 1978; Blakemore et al., 1981; Antonini and Stryker, 1998).

Similar to higher mammals, MD in rodents shifts the physiological responsiveness of neurons in the binocular zone of V1 towards the open eye, and this plasticity is confined to a well-defined CP (Dräger, 1978; Fagiolini et al., 1994; Gordon and Stryker, 1996). At least in the mouse, this is due to a rapid weakening of the deprived-eye responses, accompanied by a delayed strengthening of the open-eye responses which results from mechanisms of homeostatic plasticity (Frenkel and Bear, 2004; Kaneko et al., 2008; Cooke and Bear, 2010). Anatomical changes accompany functional plasticity in the developing visual cortex of the mouse, as they do in higher mammals (Antonini et al., 1999; Mataga et al., 2004; Oray et al., 2004).

### Treatments for amblyopia

Theoretically, the basic strategy for treating amblyopia is to provide a clear retinal image, and then to correct the OD deficit, as early as possible, during the period of visual cortex plasticity. The methods most currently used in the treatment of human amblyopia, including refractive correction applied alone or in combination with occlusion or atropine, are known as “passive methods”. Occlusion therapy with patching of the dominant eye has been widely used as the primary treatment for amblyopia (Loudon and Simonsz, 2005). The success of patching seems to correlate with the actual number of hours that the eye is patched (Loudon et al., 2002) but is also dependent on the severity of amblyopia, binocular status, fixation pattern, the age at presentation and patient compliance (Loudon et al., 2003; Stewart et al., 2005).

Atropine penalization is recognized as a valid alternative to patching for amblyopia therapy (Foley-Nolan et al., 1997; Simons et al., 1997; Pediatric Eye Disease Investigator Group, 2002). Atropine paralyzes accommodation and blurs near vision, encouraging the use of the amblyopic eye. It has been reported that atropine is as effective as patching, but that patching effects are initially faster, while atropine displays a better compliance (Pediatric Eye Disease Investigator Group, 2002). Another major difference between the two treatments is that in atropine penalization vision is binocular in the sense that the image at the fovea of the dominant (non-amblyopic) eye is degraded, while input to the amblyopic eye is not affected; in contrast, binocularity is impaired in the patching treatment.

A better strategy might be to couple passive methods with treatments in which certain tasks are prescribed to be performed by the patient: these “active” interventions could encourage a better involvement of the amblyopic eye and directly promote patient compliance, if the task is sufficiently attractive. Pleoptics is a method for visual diagnosis and training that employs monocular techniques for the detection and elimination of eccentric fixation and amblyopia: a bright ring of light is flashed around the fovea to temporarily “blind” or saturate the photoreceptors surrounding the fovea, which eliminates vision from the eccentric fixation point and forces fixation to the fovea. Typically, pleoptic treatments have to be performed several times a week in order to effectively enhance the effects elicited by occlusion therapy. Most practitioners, however, have found pleoptics to be no better than standard occlusion therapy (VerLee and Iacobucci, 1967; Fletcher et al., 1969). Another proposed active procedure was the so called CAM treatment (Campbell, 1968), consisting in a high contrast square wave grating that rotates slowly, at about one revolution per minute. The treatment was based on the findings that spatial frequency and orientation-specific filters, in the visual system, are activated by rotation. The CAM treatment was found not effective (Keith et al., 1980; Crandall et al., 1981; Tytla and Labow-Daily, 1981).

It has been established that binocular stimulation may be important for the treatment of amblyopia; indeed, animal research indicates that binocular stimulation promotes binocular cortical connections during recovery from deprivation amblyopia (Mitchell and Sengpiel, 2009). Experimental models of patching therapy for amblyopia applied to animals rendered amblyopic by a prior period of early MD indicate that the benefits of a patching therapy can be heightened when combined with critical amounts of binocular visual input each day (Mitchell and Sengpiel, 2009). Recent studies (Baker et al., 2007; Mansouri et al., 2008; Vedamurthy et al., 2008) provided new information on how signals from the amblyopic and not amblyopic eyes can impact on each other and on binocular vision (see also Mitchell and Duffy, 2014 for a recent review).

While amblyopia can often be reversed when treated early (Wu and Hunter, 2006), successful treatments are not generally possible in adults. Recently, several studies in the visual system clarified some of the mechanisms that limit plasticity to early life, showing that the adult brain is not “hardwired” with fixed neural circuits; on the contrary, following specific treatments, it can reacquire a certain degree of plasticity even well after the end of the CP (see Bavelier et al., 2010). Treatments for amblyopia in adulthood are focused on promoting cortical plasticity by reducing those factors that actively limit adult plasticity, or by exploiting endogenous permissive factors; under these favorable conditions, circuit rewiring may be facilitated in the mature brain, inducing recovery from amblyopia. Thus, several pharmacological attempts have been done to enhance adult visual cortical plasticity, acting on factors which are also thought to contribute to its developmental time course.

While, early in development, glutamatergic excitation appears to dominate cortical circuits, accumulating evidence supports a pivotal role for late-developing excitatory and inhibitory (E/I) circuit balance in the opening and successive time-course modulation of CPs. For example, the onset of visual cortical plasticity is delayed by genetic disruption of GABA synthesis or a slowing down of the maturational state of perisomatic inhibition (Hensch, 2005). Conversely, application of benzodiazepines or other treatments that accelerate GABA circuit function trigger premature plasticity (Di Cristo et al., 2007; Sugiyama et al., 2008). These manipulations are so powerful that animals of identical chronological age may be at the peak, before, or past their sensitive period, depending on how the maturational state of their GABA circuitry has been altered. The E/I circuit balance points out a possible mechanisms for enhancing recovery of function in adulthood, suggesting that a reduction of GABAergic transmission could be a crucial step for the restoration of plasticity processes in the adulthood (Hensch, 2005; Baroncelli et al., 2011). In agreement with this, a recent study showed that a pharmacological reduction of intracortical inhibition obtained through the infusion of either MPA (an inhibitor of GABA synthesis) or picrotoxin (a GABA_A_ antagonist) directly into the visual cortex reactivates OD plasticity in response to MD in adult rats (Harauzov et al., 2010).

The release of endogenous neuromodulators, such as norepinephrine, acetylcholine, serotonin, or dopamine, may also act on visual plasticity by adjusting a favorable E/I balance (Kasamatsu and Pettigrew, 1976; Bear and Singer, 1986; Kilgard and Merzenich, 1998; Bao et al., 2001; Goard and Dan, 2009). In agreement with this, it has been demonstrated that chronic treatment with the selective serotonine-reuptake inhibitor (SSRI) fluoxetine reinstates OD plasticity following MD and promotes recovery of normal visual functions in adult amblyopic animals, acting through a pronounced reduction of intracortical inhibition (Maya Vetencourt et al., 2008). Since SSRIs are approved by Food and Drug Administration, their use for treating amblyopia appears as a very promising approach. Another recent indication that neuromodulatory systems affect plasticity in adulthood comes from the demonstration that a genetic manipulation of nicotinic cholinergic transmission promotes visual cortex plasticity after the end of the CP (Morishita et al., 2010).

On the basis of recent findings indicating that environmental experience can lead to epigenetic modifications of brain chromatin status, use of epigenetic drugs can be a promising strategy also for recovery from amblyopia (Zhang and Meaney, 2010). It has been shown that a developmental downregulation of experience-dependent regulation of histone H3 and H4 acetylation is involved in the closure of the CP (Putignano et al., 2007). Recently, Silingardi et al. (2010) found that a chronic intraperitoneal administration of valproic acid, a histone deacetylase inhibitor, drives recovery from visual acuity deficits in adult rats rendered amblyopic by long-term MD.

Finally, following the demonstration that extracellular matrix penineuronal nets (PNNs) drastically limit adult brain plasticity (Pizzorusso et al., 2002), Pizzorusso et al. (2006) showed that adult chondroitinase ABC (an enzyme degrading chondroitin sulphate proteoglycans, i.e., critical components of the extracellular matrix), coupled with reverse suture (i.e., the deprivation of the previously open eye and opening of the previously deprived eye) produces a full recovery of both OD and visual acuity in amblyopic rats (replication of this finding in cats, however, has recently been shown to fail; Vorobyov et al., 2013). These authors also found that the decrease in spine density caused by long-term MD was recovered by the chondroitinase ABC treatment, suggesting that a possible mechanism underlying the recovery from amblyopia could be the formation of synaptic contacts on the newly formed spines by the inputs from the formerly deprived eye. Some of the effects elicited by chondroitinase ABC could be mediated by modifications of intracortical inhibitory circuits occurring after PNN degradation, bringing parvalbumin (PV) interneurons back to a more juvenile-like status (Hensch, 2005). Strikingly, a specific transfer of the orthodenticle homeobox 2 (Otx2) homeoprotein into GABAergic interneurons expressing PV has been shown to be a critical trigger for both the opening and closure of the CP of plasticity in the developing mouse visual cortex (Sugiyama et al., 2008). Endogenous Otx2 is captured by specific binding sites in PNNs placed on the surfaces of PV cells, with a short aminoacidic domain containing an arginine-lysine doublet, called RK peptide, directly mediating Otx2 binding to PNNs (Beurdeley et al., 2012). Chondroitinase ABC reduces the amount of endogenous Otx2 in PV cells, and infusion of RK peptide disrupts endogenous Otx2 localization to PV cells and PNN expression, leading to restoration of binocular vision in adult amblyopic mice (Beurdeley et al., 2012).

A better strategy for amblyopia treatment would be that to induce an endogenous recapitulation of the brain states that promote plasticity in a non-invasive but targeted manner. Amblyopic rats subjected to complete visual deprivation by dark exposure for 10 days recover significant vision once allowed to see binocularly, acting through a modulation of the balance between excitation and inhibition (He et al., 2007). However, translation of this treatment to humans is debatable as the proportional length of dark exposure required is likely to be quite long. A more promising approach is environmental enrichment (EE). EE is an experimental protocol specifically designed to investigate the influence of the environment on brain and behavior (Rosenzweig and Bennett, 1996; van Praag et al., 2000; Diamond, 2001; Sale et al., 2014). “Enriched” animals are reared in large groups in wide cages where a variety of toys, tunnels, nesting material and stairs are present and changed frequently. Thus, EE aims at optimizing environmental stimulation by providing the animals with the opportunity to attain high levels of voluntary physical activity, spontaneous exploration, cognitive activity and social interaction. We showed that EE promotes a complete recovery of visual acuity and OD in adult amblyopic animals (Sale et al., 2007). Recovery of plasticity was associated with a marked reduction of GABAergic inhibition in the visual cortex, as assessed by brain microdialysis. Moreover, a decreased cortical inhibition was demonstrated also at the synaptic level, using the *in vitro* paradigm of LTP of layer II–III field potentials induced by theta-burst stimulation from the white matter (WM–LTP). The WM–LTP is normally not present in the adult as a result of the maturation of inhibitory circuits (Kirkwood and Bear, 1994; Huang et al., 1999), but it can be restored if GABA-mediated inhibition is reduced (Artola and Singer, 1987; Kirkwood and Bear, 1994). Notably, the ability of the cortex to undergo WM-LTP was fully reinstated in the visual cortex of EE adult rats (Sale et al., 2007). The reduction of cortical inhibition in EE rats was also paralleled by an increased expression of the neurotrophin BDNF and a lower density of PNNs in the visual cortex contralateral to the recovering (previously amblyopic) eye.

## Visual perceptual learning

Perceptual learning (PL) is currently considered one of the most promising active strategies for treating amblyopia in adulthood.

### Definition and variety of the phenomenon

Perceptual learning is the improvement in performance on a variety of simple sensory tasks, following practice. In visual perception, such tasks, often called discrimination tasks, involve identifying small differences in simple visual attributes, such as position, orientation, texture or shape.

Visual PL has been documented in a wide range of perceptual tasks: stimulus orientation discrimination (Vogels and Orban, 1985; Shiu and Pashler, 1992; Schoups et al., 1995; Matthews and Welch, 1997; Matthews et al., 1999), motion direction discrimination (Ball and Sekuler, 1982, 1987; Ball et al., 1983; Matthews and Welch, 1997), discrimination of differences in the waveforms of two grating stimuli (Fiorentini and Berardi, 1980, 1981; Berardi and Fiorentini, 1987), detection of visual gratings (De Valois, 1977; Mayer, 1983); texture discrimination (Karni and Sagi, 1991, 1993; Ahissar and Hochstein, 1996); discrimination of changes in spatial frequency within simple or complex plaid patterns (Fine and Jacobs, 2000); ability to detect small differences in the depth of two targets (Fendick and Westheimer, 1983; Westheimer and Truong, 1988); ability to perceive depth in random-dot stereograms (Ramachandran and Braddick, 1973); ability to discriminate between 10 band-pass Gaussian filtered noise texture (Gold et al., 1999a); object (Furmanski and Engel, 2000) and face recognition (Gold et al., 1999b). Training can improve the discrimination of small differences in the offset of two lines (Vernier acuity), even though initial thresholds are already in the hyperacuity range (McKee and Westheimer, 1978). In addition, a number of studies indicate that visual acuity can improve with practice also in hyperacuity tasks (Bennett and Westheimer, 1991; Poggio et al., 1992; Fahle and Edelman, 1993; Beard et al., 1995; Saarinen and Levi, 1995; Fahle and Morgan, 1996).

An important component of visual PL is the rate at which learning occurs. For some visual tasks, the learning effect has been found to take place within an hour or two (Fiorentini and Berardi, 1980, 1981; Shiu and Pashler, 1992; Fahle et al., 1995; Liu and Vaina, 1998). In some studies, learning is practically complete after a few hundreds of trials (Fiorentini and Berardi, 1980, 1981), showing fast saturation. For other tasks, there is an initial fast saturating phase of learning, which is then followed by a slow phase where the performance continues to improve from one daily session to the next one, until a stable optimal level is reached (Karni and Sagi, 1991). Interestingly, Karni and Sagi (1993) found that an improvement between sessions occurs only if the two sessions are separated by at least 68 h, suggesting the existence of a consolidation period.

Visual PL shows a high specificity for the features of the stimuli used in the task. Many studies reported that the visual performance is typically improved on test trials that use the same stimuli as those used during training, and that the achieved performance often returns to baseline levels when test trials adopt even mildly different stimuli. A specificity of learning has been found for the orientation of lines and gratings (Ramachandran and Braddick, 1973; McKee and Westheimer, 1978; Fiorentini and Berardi, 1980, 1981; Karni and Sagi, 1991; Poggio et al., 1992; Fahle and Edelman, 1993; Schoups et al., 1995) or the direction of motion (Ball and Sekuler, 1982, 1987), and for the retinal location of the stimuli used in the learning procedure (Fiorentini and Berardi, 1981; Ball and Sekuler, 1987; Karni and Sagi, 1991; Shiu and Pashler, 1992; Schoups et al., 1995). Fiorentini and Berardi (1980) found that practice improved discrimination between complex gratings, and that the achieved improvement did not transfer to stimuli rotated by 90°.

In most cases, visual PL is not restricted to the eye employed, i.e., if the training process is monocular, learning transfers completely or partially to the untrained eye (Fiorentini and Berardi, 1981; Ball and Sekuler, 1982; Beard et al., 1995; Schoups et al., 1995); this indicates that the learning process occurs more centrally with respect to the site where the inputs from the two eyes converge. Texture discrimination is an exception in this respect, showing little interocular learning transfer (Karni and Sagi, 1991; Schoups and Orban, 1996).

### Neural changes underlying visual perceptual learning

The selectivity of visual PL for basic attributes of the stimuli, such as orientation (Ramachandran and Braddick, 1973; McKee and Westheimer, 1978; Fiorentini and Berardi, 1980, 1981; Karni and Sagi, 1991; Poggio et al., 1992; Fahle and Edelman, 1993; Schoups et al., 1995), motion direction (Ball and Sekuler, 1982, 1987) and even retinal location (Fiorentini and Berardi, 1981; Ball and Sekuler, 1987; Karni and Sagi, 1991; Shiu and Pashler, 1992; Schoups et al., 1995), suggests the involvement of early stages in cortical visual processing, where neurons have relatively small receptive fields (RFs), are selective for stimulus features such as orientation, size, chromatic properties and direction of motion, and the visual topography is most precisely mapped.

The specificity of learning for basic visual features does not imply that the representations of learning occur only in the early stage of the visual system. Cortical changes associated with PL can also occur in intermediate visual stages. Changes have been reported in the tuning properties of cells in V4 in monkeys trained in an orientation discrimination task, whereas no such tuning changes were observed in V1 (Ghose et al., 2002; Yang and Maunsell, 2004). Yang and Maunsell (2004) were the first to demonstrate that PL modifies basic neuronal response properties at an intermediate middle level of visual cortical processing (V4). They found that an orientation discrimination task changes the response properties of V4 neurons: after training, neurons in V4 with RFs overlapping the trained location had stronger responses and narrower orientation tuning curves than neurons with RFs in the opposite, untrained hemifield. Moreover, neurons with preferred orientations, nearby the trained one, show the most relevant modifications.

The idea that changes associated with PL occur exclusively in early or intermediate visual areas has been challenged by the results of neurophysiological studies in monkeys (Chowdhury and DeAngelis, 2008; Law and Gold, 2008). In one of these studies (Law and Gold, 2008), learning to evaluate the direction of visual motion did not change the responses of cells in the middle temporal area (MT), a region highly responsive to motion, but did change the responses of cells in the lateral intraparietal area (LIP), a region that is known to represent the transformation of visual motion signals into responses by saccadic eye movements. However, PL-induced changes in MT have also been reported. For example, Zohary et al. (1994) studied the simultaneous activity of pairs of neurons recorded with a single electrode in MT while monkeys performed a direction discrimination task, exploring the relationship between inter-neuronal correlation and behavioral and stimulus parameters. They reported that spike counts from adjacent neurons were noisy and only weakly correlated, but that even this small amount of correlated noise could affect signal pooling, suggesting a relationship between neuronal responses and psychophysical decisions.

Attention exerts a significant influence on many types of PL. Some studies found that a conscious effort to direct focused attention plays an important role in gating visual plasticity, suggesting that focused attention must be directed to a feature in order to be learned (Shiu and Pashler, 1992; Ahissar and Hochstein, 1993; Herzog and Fahle, 1998; Gilbert et al., 2001; Schoups et al., 2001). Little or no transfer learning has been reported between two tasks that used the same visual stimuli but involved judgments on different stimulus attributes (either orientation of local elements or global shape) (Ahissar and Hochstein, 1993). It has also been demonstrated that the discrimination of orientation of lines did not improve when a non attended feature was presented (brightness rather than orientation of the line) (Shiu and Pashler, 1992). Furthermore, an electrophysiological study in monkeys demonstrated that PL resulted in the sharpening of orientation tuning curves only for V1 cells with RFs overlapping to the spatial location of the training task (Schoups et al., 2001). Additionally, it has been proved that PL is task-dependent, indeed there is no transfer in learning of a particular feature between tasks involving similar stimuli but using a different procedure (Li et al., 2004; Huang et al., 2007).

However, evidence from studies of “task-irrelevant” learning shows that PL can also occur in the absence of focused attention to the learned feature (Watanabe et al., 2001; Seitz and Watanabe, 2003; Nishina et al., 2007). A follow-up study demonstrated that this task-irrelevant kind of learning was highly specific for local motion of the stimuli, as opposed to the global motion, and that learning was retained for months after training (Watanabe et al., 2002). These findings indicate that focused-attention is not necessary for PL, but task-irrelevant learning might not occur simply as a result of exposure to a stimulus. Seitz and Watanabe (2005) proposed a model for task-irrelevant learning that can also explain task-relevant learning. Based on this model, PL occurs through the coincidence of diffusive signals driven by a task activity (reinforcement signals) and signals induced by the presentation of a stimulus (stimulus-driven signals). In this model, the task target induces both reinforcement signals and stimulus-driven signals, thus when task-irrelevant target and reinforcement signal interact with an appropriate temporal relationship, learning of task-irrelevant features can occur.

Gilbert et al. (2009) proposed that PL is associated with long-term modification of cortical circuits. In this view, top-down influences of attention, expectation and the nature of the perceptual task interact with experience-dependent modification processes at the early level of the visual system. Both anatomical and physiological data show that V1 neurons can integrate information over an area much larger than their RFs measured with oriented line, and that this functional property is due to a large extent to the axonal arbors of cortical pyramidal cells (Gilbert and Wiesel, 1979, 1983; Rockland and Lund, 1982; Stettler et al., 2002). The horizontal connections link orientation columns with similar orientation preference (Stettler et al., 2002), and account for the majority of the inputs that neurons receive, with over 76% of excitatory inputs arising from outside their resident hypercolumn (Stepanyants et al., 2009). Thus, these long range connections provide neurons with selectivity for features more complex than the ones predicted from their RFs, endowing neurons with context-dependent responses.

### Cellular mechanisms underlying perceptual learning

Despite recent progress in localizing the visual areas involved in PL, elucidation of the underlying mechanisms at the cellular level remains a challenge. Learning is supposed to rely on changes in neuronal circuits in brain areas specific for the practiced task, leading to long-lasting modifications in synaptic efficacy (synaptic plasticity). While the notion that synaptic plasticity underlies learning is widely accepted for declarative memory processes mediated by temporal lobe areas or for implicit forms of memory such as classical conditioning (Kandel, 2009), the specific role of synaptic plasticity in PL, a form of implicit memory, remains unclear. It has been shown that skill motor learning leads to long-lasting synaptic plasticity changes in the primary motor cortex (M1; Rioult-Pedotti et al., 2000) and, in the visual system, changes in V1 activity have been documented following visual PL both in monkeys and humans (e.g., Schoups et al., 2001; Li et al., 2008; Yotsumoto et al., 2008). At present, however, there is no conclusive evidence for the presence of synaptic plasticity phenomena in V1 in correlation with visual PL.

Several possible cellular mechanisms have been proposed to account for the effects of PL. One possibility is that the number of neurons representing the learned stimulus increases after training; this mechanism has been found mainly in the auditory (Recanzone et al., 1993) and somatosensory (Recanzone et al., 1992) cortex. In the visual system, PL appears to be mediated primarily by changes in the response strength or tuning of individual neurons, rather than large-scale spatial reorganization of the cortical network, as found in the auditory and somatosensory systems.

Schoups et al. (2001) demonstrated that changes in V1 orientation tuning accompany improved performance in orientation discrimination in adult monkeys. However, they did not find an increase in the proportion of neurons tuned to the trained orientation, but they reported an increase in the slope of the tuning curve at the trained orientation for neurons with preferred orientations lying between 12° and 20° of the trained one. The authors suggested that learning is correlated with changes in tuning curves of specific group of neurons that are most sensitive to small changes near the trained orientation, and, thus, that are relevant for detecting an orientation difference. Therefore, sharpening of tuning curves of cells, whose steepest parts of tuning curves coincide with the trained attribute, can improve discrimination of trained features, leading to more selective and less overlapping cortical representations. On the contrary, Ghose et al. (2002) found that PL caused only a small reduction in the response amplitude of V1 and V2 cells tuned to the trained orientation, suggesting that the psychophysical change is mediated by top-down influence for the trained task, and not by an improved neural representation of orientation in early visual areas.

Very few studies involving visual PL have been performed in rodents. Stimulus-induced vision restoration (visual training) has been proposed to be achievable in a plethora of different types of visual field impairments due to retinal or brain damage (e.g., stroke, amblyopia, age-related macular degeneration) (reviewed in Sabel et al., 2011). With the declared aim to investigate whether cortical plasticity might depend on the temporal coherence of visual stimuli, Matthies et al. (2013) showed that substantial OD plasticity can be triggered in adult mice visually stimulated by the presentation of moving square wave gratings during a period of MD, even within very short periods of time (2 days). Frenkel et al. (2006) previously described a different form of experience-dependent response enhancement (called stimulus-selective response potentiation, SRP) in the visual cortex of awake mice. They found that repeated exposure to grating stimuli with specific orientation results in a potentiated response evoked by the test stimulus. The long-lasting enhancement of visual responses increased gradually over the training sessions, was specific for the orientation of the grating stimuli used, and occurred in both juvenile and adult mice. Moreover these authors reported that SRP induced through one eye did not transfer to the contralateral eye, suggesting the involvement of early stages of visual processing. While in primates the neural substrate involved in PL may have a deep dependence on training specificity, in rodents the relationship between learning and neural changes may be simpler. The effects observed by Frenkel et al. (2006) are consistent with a cortical change induced by PL, even if the stimulus-induced plasticity of SRP is not a form of perceptual learning, since no specific task was required. Interestingly, this cortical modification is more similar to the increase in fMRI response obtained in the human visual cortex after PL (i.e., Furmanski et al., 2004) compared with results obtained with single-unit recordings in monkey V1 (i.e., Schoups et al., 2001). Moreover, visual neurons can respond to non-visual inputs if they are paired with visual stimuli in a learning task: after training rats in a task that associates visual stimuli with a subsequent reward, Shuler and Bear (2006) found that a significant proportion of neurons show activity that correlated with the time in which the reward was given.

Given that PL is able to promote neural plasticity in early visual areas, possibly determining the potentiation of the visual connections active during learning, it could be exploited to facilitate recovery from conditions in which deficits in a set of visual neural connections lead to visual impairments. In the last two decades, there has been a progressive increase in studies that have tested and developed visual rehabilitation programs based on PL. We shall now discuss the possible application of PL for amblyopia treatment.

## Perceptual learning as a potential treatment for amblyopia

PL has been shown to remarkably improve visual functions in amblyopia on a wide range of tasks, including Vernier acuity (Levi and Polat, 1996; Levi et al., 1997), positional acuity (Li and Levi, 2004; Li et al., 2005, 2007), contrast sensitivity (Polat et al., 2004; Zhou et al., 2006; Huang et al., 2008), and first-order and second-order letter identification (Levi, 2005; Chung et al., 2006, 2008). While practicing each of these tasks results in improved visual performance, the high specificity of PL and the lack of transfer of PL effects to untrained orientations (Levi and Polat, 1996; Levi et al., 1997; Li and Levi, 2004) or from a Vernier acuity task to a detection task (Levi and Polat, 1996; Levi et al., 1997) can reduce its therapeutic value in the treatment of amblyopia. However, it has been shown that in various tasks (e.g., vernier acuity, position discrimination and contrast sensitivity) PL appears to transfer, at least in part, to improvements in visual acuity measured, for example, with the Snellen chart (Levi and Polat, 1996; Levi et al., 1997; Li and Levi, 2004; Polat et al., 2004; Zhou et al., 2006; Huang et al., 2008). Additionally, other impaired visual functions, such as stereoacuity and visual counting (Li and Levi, 2004; Li et al., 2007), improved with PL as well as visual acuity. Importantly, in adults with normal vision the improvements obtained through PL last for months, even for years (e.g., Karni and Sagi, 1993), and Li et al. (2004) reported that the improvement in visual acuity in the amblyopic eye induced by position discrimination training was long-lasting (from 3 to 12 months). Moreover, the effects in the improvement in visual acuity was present 12 months past the end of learning (Polat et al., 2004) and, in few cases, with a level of retention of approximately 90% (Zhou et al., 2006).

**Figure 1:**
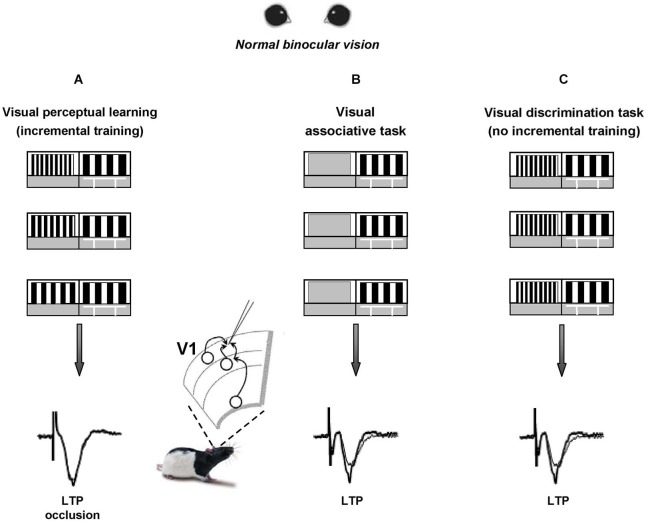
**Visual perceptual learning induces long-term potentiation in the primary visual cortex**. A modified version of the visual water box task is used to induce visual perceptual learning (PL) in a group of adult rats (**panel A**) that are first trained to distinguish a low 0.117 cycles per degree (c/deg) spatial frequency (SF) grating (reference grating) from a 0.712 c/deg SF grating (test grating) and then learned to distinguish the two gratings when they became more and more similar to each other. Two groups of control animals are trained to either distinguish the reference grating from a homogeneous gray (**panel B**) or to distinguish a low SF vs. a never changing high SF panel (**panel C**, thus lacking the incremental training). After training, LTP from layer II-III of V1 slices is occluded in PL animals compared to controls, at the level of both vertical and horizontal connections.

We recently reported that visual PL induces long-term potentiation (LTP) of intracortical synaptic responses in rat V1 (Sale et al., 2011). To elicit visual PL, we first trained a group of adult animals to practice in a forced-choice visual discrimination task that requires them to distinguish between two vertical gratings differing only for their spatial frequency; then, we made the two stimuli progressively more similar to each other (Figure 1A), until the animal performance reached a steady plateau. This task requires activation of V1 circuits, as indicated by the strong selectivity of PL for the orientation of gratings employed during training (Sale et al., 2011). Control animals only learned an association task, i.e., they were only required to discriminate between a grating and a homogeneous gray panel (Figure 1B), matching the overall swim time and number of training days in the water maze with those of PL rats.

Within 1 h from the last discrimination trial, LTP from layer II-III of V1 slices appeared occluded in PL animals compared to controls (Figure 1), both when testing its inducibility in vertical connections (stimulating electrode placed in layer IV) and when stimulating at the level of horizontal connections (stimulating electrode placed in layer II/III). Moreover, a significant shift toward increased amplitude of fEPSPs was found in the input/output curves of trained animals compared to controls (Sale et al., 2011). Thus, the data fulfill two of the most commonly accepted criteria used to relate LTP with learning, i.e., occlusion and mimicry, demonstrating that the improvements displayed by PL rats in discriminating visual gratings of progressively closer spatial frequencies can be explained in terms of long-term increments of synaptic efficacy in V1, the same cortical area at work during perception. This is consistent with the critical role for LTP in mediating learning processes previously reported in other brain areas such as the amygdala, the hippocampus and the motor cortex (Rogan et al., 1997; Rioult-Pedotti et al., 1998; Whitlock et al., 2006).

**Figure 2:**
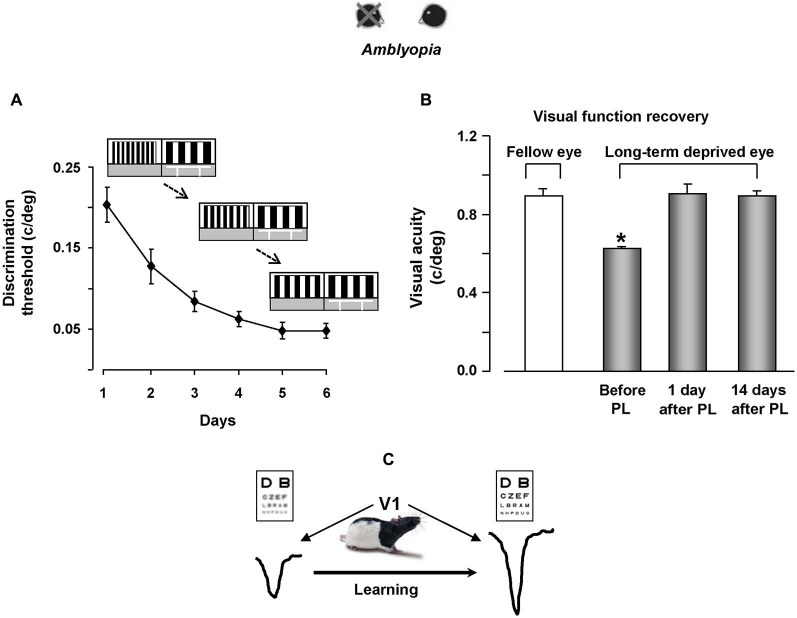
**Visual perceptual learning promotes vision recovery in adult amblyopic rats**. **(A)** Improvement of discrimination threshold in adult amblyopic rats performing the visual PL task. The threshold, calculated as the minimum spatial frequency difference between the reference and the test gratings discriminated (MDSFD), decreases significantly with the training days. **(B)** Behavioral measure of visual acuity recovery in rats subjected to visual PL. Visual acuity of both the long-term deprived and the open eye is measured using the visual water box task. At the end of the PL procedure, visual acuity of the previously deprived eye is not different from that of the fellow eye, an effect outlasting the end of the treatment by at least 2 weeks. **(C)** We propose a model in which recovery of visual functions for the long-term deprived eye is driven by potentiation of synaptic transmission elicited by visual PL.

Since a potentiation of synaptic transmission might help the recovery process of visual responses for the long-term deprived eye, practice with visual PL through the amblyopic eye is expected to favor a functional rescue in amblyopic animals. In agreement with evidence on human subjects, a marked recovery of visual functions was evident in amblyopic rats subjected to visual PL (Baroncelli et al., 2012; Figures 2A,B), while no recovery occurred in two control groups in which the treatment did not induce LTP in V1, i.e., in rats that only learned the associative visual task and in animals that were trained only until the first step of the discrimination procedure between the test and the reference grating (Figure 1C), without proceeding further with a progression of finer discrimination trials (Baroncelli et al., 2012). Since these two control groups were matched to the animals trained in the PL procedure in terms of overall swim time in the water maze, their lack of recovery clearly indicates that the physical exercise component associated with our PL procedure does not contribute to the recovery of vision. This conclusion could seem at odd with the results showing a full recovery of both OD and visual acuity in adult amblyopic rats subjected to a period of intense physical exercise in a running wheel (Baroncelli et al., 2012). However, the lack of recovery found in the two control groups could be due to the purely forced nature of the exercise imposed to them: while running rats performed a form of totally voluntary movement, physical activity in the water maze is necessarily forced and artificially imposed. Several lines of evidence suggest that forced exercise and voluntary exercise exert different effects on brain and behavior. For example, forced and voluntary exercise differentially affect monoamine neurotransmitters (Dishman et al., 1997), hippocampal PV expression (Arida et al., 2004), hippocampal brain-derived neurotrophic factor and synapsin-1 expression (Ploughman et al., 2005), longevity and body composition (Narath et al., 2001), taste aversion learning (Masaki and Nakajima, 2006) and open-field behavior (Burghardt et al., 2004). On the other hand, the marked rescue of visual abilities obtained in PL rats underscores the importance and effectiveness of visual practice and incremental training in driving recovery from amblyopia.

The recovery effect achieved by trained rats persisted for quite a long time, outlasting the end of the treatment by at least 14 days (Figure 2B), corresponding to 20 months or more in the timescale of human life.

Our results also underscored a transfer effect in two distinct manners: first, the recovery of visual acuity was not limited to stimuli of the same orientation than that used during the PL procedure, but was also present for orthogonal stimuli; second, even if rats practiced in discriminating visual gratings in the 0.1–0.6 c/deg range, they displayed a discrimination improvement in a range of higher spatial frequencies, with final VA values in the range of 0.9–1.0 c/deg (Baroncelli et al., 2012).

One of the clearest advantages in the use of animal models of human pathologies is the possibility to investigate the underlying molecular mechanisms. Recovery of visual abilities in PL animals was accompanied by a robust decrease of the inhibition-excitation balance, crucially involved in the regulation of plasticity both during development and in adulthood (Hensch, 2005; Morishita and Hensch, 2008; Spolidoro et al., 2009; Harauzov et al., 2010; Sale et al., 2010; Baroncelli et al., 2011; van Versendaal et al., 2012; Kuhlman et al., 2013). These results provide the first evidence that PL is associated with reduced inhibition/excitation balance in V1. The relative strength of excitatory and inhibitory connections has been suggested to be impaired during development in amblyopic human subjects and cortical over-inhibition could underlie the degradation of spatial vision abilities (Polat, 1999; Levi et al., 2002; Wong et al., 2005). Repetitive transcranial magnetic stimulation, which increases cortical excitability, transiently improves contrast sensitivity in adult amblyopes, likely acting on the excitation/inhibition balance (Thompson et al., 2008). The reduction of intracortical inhibition could be downstream from the modulation of neuromodulatory release, such as the potentiation of serotonin transmission: it has been demonstrated that the infusion of an inhibitor of 5-HT can counteract the decrease in number of GAD67 expressing cells induced by EE (Baroncelli et al., 2010), and, moreover, it has been reported that serotonin can inhibit GABA release via a presynaptic mechanism, probably by regulating the availability of transmitter vesicles (Wang and Zucker, 1998).

As stated previously, we found that PL increases the synaptic strength of intracortical connections in V1. Li and Gilbert suggested a mechanism for PL based on the interaction between feedback and horizontal connections (Gilbert et al., 2009; Gilbert and Li, 2013). In this view, visual responses are dependent on the behavioral context, according to the perceptual task performed, and the contextual influence can be mediated by horizontal connections within V1 (Gilbert et al., 2009), since these long-range connections provide neurons with selectivity for complex features (Gilbert and Wiesel, 1979; Li and Gilbert, 2002; Stettler et al., 2002). Thus, with PL practice, it is possible that the horizontal connections could mediate a synchronized output response for the stimulus used in the task, by recruiting neurons that show selectivity for similar orientation and that are engaged in the perceptual task. It is known that synchronized electrical activity in gamma frequency band is correlated with conscious processing of sensory stimuli and higher cognitive functions such as attention and memory and that these gamma oscillations can occur locally within a brain region or distributed in a brain-wide manner among different regions (Gray and Singer, 1989; Gray et al., 1989; Tiitinen et al., 1993; Desmedt and Tomberg, 1994; Gray and McCormick, 1996; Miltner et al., 1999; Tallon-Baudry and Bertrand, 1999; Fries et al., 2001, 2002; Brosch et al., 2002; Laurent, 2002; Sederberg et al., 2003; Gruber et al., 2004; Tallon-Baudry et al., 2005; Axmacher et al., 2006; Jokisch and Jensen, 2007; Melloni et al., 2007). In the visual system, Gray and Singer (1989) recorded a gamma oscillatory field potential that was strongly correlated with visual stimuli specific for the orientation preference, demonstrating that neurons within a given orientation column show stimulus-dependent selectivity. Moreover, the same authors demonstrated that a synchronized activity was present also across the orientation columns: they found that neural responses were selective for feature of visual stimulus and that the neurons involved are located in superficial layers, thus the likely candidates for the synchronization activity are horizontal connections (Gray et al., 1989; Engel et al., 1990; Gray and McCormick, 1996).

The top-down influence could play a significant role in PL by selecting an appropriate contextual influence, mediated by long-range horizontal connections within each cortical area (Gilbert et al., 2009). The majority of V1 cortical output is sent to V2, and most of the feedback connections come from V2, even if V1-V2 circuitry is more complex than previously thought (Sincich and Horton, 2005), with the recent demonstration that V2 exerts a modulatory effect on V1 through feedback projections that end in layer IV of V1 (De Pasquale and Sherman, 2013). Furthermore, V1 receives feedback connections from other visual areas, including V4, MT, and the inferotemporal cortex, and it has been also suggested that connections from higher- to lower-order visual areas might be mediated by a cortex-to-thalamus-to-cortex pathway (Sherman, 2005).

## Conclusions

These findings can be used to depict a general theoretic model concerning the cellular processes underlying visual PL in V1. Such a model requires taking into account the strategy employed by the trained rats, which practiced the discrimination between gratings while they were highly motivated to find the hidden platform. In this process, an involvement of extra-V1 projections is very likely to take place. An interaction between the appropriate V1 intrinsic connections and the top-down feedback signals associated with the expectations of the behavioral task is a possible explanation for the induction of a potentiation process. The strong excitatory projections received by V1 and coming from higher order areas like V2, the secondary motor cortex, the temporal association cortex and the perirhinal cortex (Coogan and Burkhalter, 1993; Bai et al., 2004) could carry information about the animal’s behavioral and motivational state, setting the early visual areas in a specific working mode that allows the comparison of already stored representations with new bottom-up information concerning the stimulus characteristics (Gilbert et al., 2009; Gilbert and Li, 2013). This loop may have a fundamental role in PL. It is likely that the event represented by the finding of the submerged platform is associated with a given spatial frequency value and that this association forms the basis for further comparisons performed during subsequent expositions to the new spatial frequencies of the test grating. It is admissible that a simultaneous firing of higher centers’ projections carrying top-down signals and intrinsic V1 neurons selective for the stimulus parameter may lead to the induction of a synaptic potentiation process of V1 connections which eventually underlies the improvement in sensory discrimination (Figure 2C).

## Conflict of interest statement

The authors declare that the research was conducted in the absence of any commercial or financial relationships that could be construed as a potential conflict of interest.
